# Antimalarial drug discovery: progress and approaches

**DOI:** 10.1038/s41573-023-00772-9

**Published:** 2023-08-31

**Authors:** Jair L Siqueira-Neto, Kathryn J. Wicht, Kelly Chibale, Jeremy N. Burrows, David A. Fidock, Elizabeth A. Winzeler

**Affiliations:** 1 University of California, San Diego, La Jolla, CA, USA.; 2 Holistic Drug Discovery and Development (H3D) Centre, University of Cape Town, Rondebosch, South Africa.; 3 South African Medical Research Council Drug Discovery and Development Research Unit, Department of Chemistry and Institute of Infectious Disease and Molecular Medicine, University of Cape Town, Rondebosch, South Africa.; 4 Medicines for Malaria Venture, Geneva, Switzerland.; 5 Department of Microbiology and Immunology and Center for Malaria Therapeutics and Antimicrobial Resistance, Division of Infectious Diseases, Department of Medicine, Columbia University Irving Medical Center, New York, NY, USA.

## Abstract

Recent antimalarial drug discovery has been a race to produce new medicines that overcome emerging drug resistance, whilst considering safety and improving dosing convenience. Discovery efforts have yielded a variety of new molecules, many with novel modes of action, and the most advanced are in late-stage clinical development. These discoveries have led to a deeper understanding of how antimalarial drugs act, the identification of a new generation of drug targets, and multiple structure-based chemistry initiatives. The limited pool of funding means it is vital to prioritize new drug candidates. They should exhibit high potency, a low propensity for resistance, a pharmacokinetic profile that favours infrequent dosing, low cost, preclinical results that demonstrate safety and tolerability in women and infants, and preferably the ability to block *Plasmodium* transmission to *Anopheles* mosquito vectors. In this Review, we describe the approaches that have been successful, progress in preclinical and clinical development, and existing challenges. We illustrate how antimalarial drug discovery can serve as a model for drug discovery in diseases of poverty.

## Introduction

Malaria has had an outsized impact on human genetics and human history. The disease, spread by mosquitoes and caused by several protozoan *Plasmodium* species, is predicted to have killed more than 300 million people in the twentieth century^1^. Symptoms are generally non-specific and can include fever, headache, malaise, weakness, gastrointestinal distress, dizziness, confusion, disorientation or even coma. Malaria causes substantial morbidity and mortality and leads to an estimated US$ 12 billion annual loss in economic activity in sub-Saharan Africa, where 95% of cases and 96% of deaths occur. The social impact on families, education, the workplace and communities is immense^2^. Contrary to earlier optimistic predictions that malaria might be eradicated by 2030 (ref. 3), case numbers have been increasing for several years: in 2021, there were an estimated 247 million malaria cases and 619,000 malaria deaths, which is 33 million more cases and 181,000 more deaths than in 2015 (ref. 4). Reasons for this increase could include biological factors such as the emergence of parasite and mosquito vector resistance to drugs and insecticides, respectively; environmental factors such as climate change and changes in vector distribution; and operational factors, including donor fatigue, improved diagnosis and tracking of disease, counterfeit drugs, economic crises, and disruptions to government and community health services from the COVID-19 pandemic.

In contrast to many viral diseases, a malaria infection provides limited immunity against subsequent reinfections. It is not understood whether this is because there is an incomplete immune response or because of the great diversity of genetic variants. Nevertheless, the disease often becomes less severe with repeated infections. Despite the challenges associated with creating a vaccine against a eukaryotic organism with 5,500 genes and well-honed mechanisms of immune evasion (such as antigenic variation), scientists have succeeded in designing vaccines that can reduce disease severity and prevent deaths. Although the introduction of the Mosquirix (RTS,S/AS01) vaccine in 2021, approved by the WHO, was a breakthrough in malaria control, none of the vaccine candidates provides sterilizing immunity^5,6^. One of these vaccine candidates is R21/Matrix-M, which recently reached the WHO goal of 75% efficacy in protecting children for 12 months in limited areas of seasonal malaria^7^. Of note, almost all participants in clinical vaccine studies received standard measures, including insecticide-treated bed nets and seasonal malaria chemoprevention (SMC) drug combinations. Therefore, a promising strategy for malaria control is the combination of a pre-erythrocytic stage malaria vaccine with an effective chemopreventive therapy^8^. Malaria has traditionally been controlled with chemotherapy^9,10^. In fact, small molecules were being used to control parasite growth long before the development of antibiotics. The quinoline ring and endoperoxide bridge found in quinine and artemisinin (ART), respectively, are part of natural products contained in extracts of cinchona bark (quinine) and sweet wormwood (*Artemisia annua*), which have been used for centuries as herbal remedies to treat fevers. Antimalarial drugs inspired by these molecules and discovered in the twentieth century continue to save millions of lives annually. However, as described in this Review, some of these drugs have liabilities, and both emerging and well-documented resistance mean that there is a continued need to refill the development pipeline with new and improved candidate drugs as parasites acquire resistance to older medicines.

This Review focuses on the history and current landscape of small-molecule antimalarial therapies and provides perspectives on future new treatments.

## Current antimalarial therapies

Malaria is an acute disease that can evolve quickly and be lethal in days; therefore, patients suspected of being infected should receive an urgent diagnosis and, if confirmed, prompt treatment. The therapies most used for uncomplicated *Plasmodium falciparum* malaria are the ART-based combination therapies (ACTs); in particular, artemether–lumefantrine (AL), which constitutes 75% of the African market, and artesunate–amodiaquine as the second-line choice, with 24% of the market share. Other much less used options include combinations of dihydroartemisinin–piperaquine (DHA–PPQ), atovaquone–proguanil (Malarone), and quinine with doxycycline or clindamycin. Treatments for severe malaria require injections, and the preferred drug is intravenous artesunate, although quinine can also be used. Chloroquine in combination with either primaquine or tafenoquine is the treatment option for radical cure of *Plasmodium vivax*, which is present in the American continent, East Africa and Southeast Asia. Intermittent preventive treatment in pregnancy with sulfadoxine–pyrimethamine (SP) involves a monthly dose from the second trimester onwards and is the standard of care across Africa. SMC with SP and amodiaquine involves a monthly administration of SP–amodiaquine (single-dose SP and amodiaquine once a day for 3 days) to children from 6 months to 5 years of age (in certain countries up to 10 years) and is used in the African Sahel, where malaria is highly seasonal and SP resistance is limited. Notably, SMC has been significantly scaled up in the past decade, with 45 million children receiving it across 15 African countries in 2021 compared with 0.2 million in 2012 (ref. 4). All these medications have direct antiparasitic activity and the choice of therapy depends on factors such as the severity of the symptoms, patient age and other risk factors, including pregnancy or compromised immunity.

## Next generation of antimalarial therapies

**Fig. 1 |:**
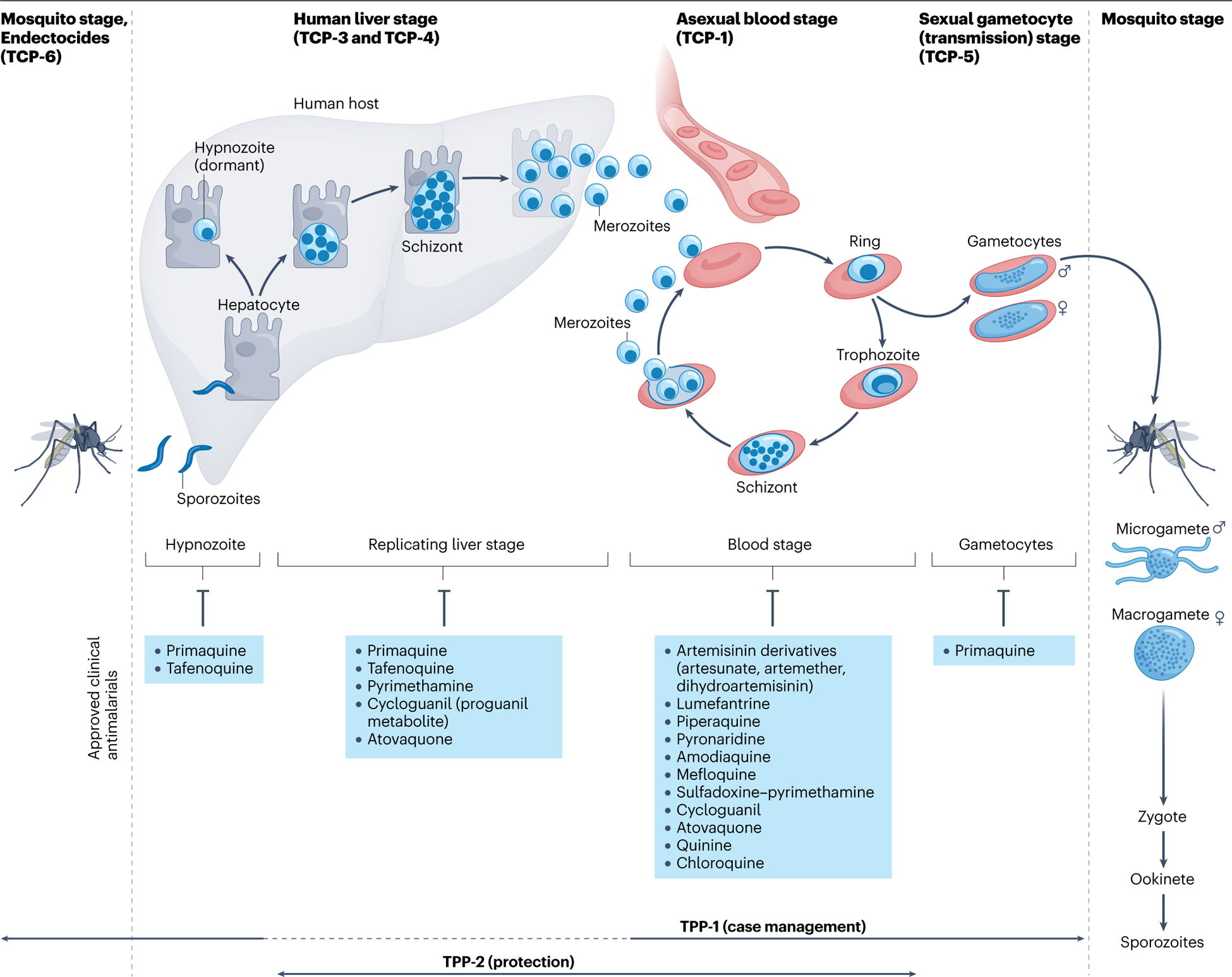
Malaria therapies in the context of the *Plasmodium* parasite lifecycle. During its blood meal on a human host, the female *Anopheles* species mosquito delivers sporozoites that enter the skin and subsequently target liver cells (hepatocytes). Within the liver, the parasite matures into schizonts (or dormant hypnozoites for *Plasmodium vivax* and *Plasmodium ovale*), followed by bursting merozoites that invade erythrocytes (the asexual blood stage). The parasite forms a ring in each erythrocyte that grows into a trophozoite with an acidic digestive vacuole that digests haemoglobin to liberate peptides and amino acids required for protein synthesis. These parasites mature into multinucleated schizonts, from which thousands of merozoites rupture, allowing for rapid replication as they reinvade new red blood cells. Small percentages of merozoites differentiate into male and female gametocytes to initiate the sexual transmission stage, where parasites are ingested by a female mosquito during its blood meal to continue the cycle. Antimalarial drugs that act on the asexual blood stage are categorized as target candidate profile 1 (TCP-1), whereas molecules active against liver-stage hypnozoites (*P. vivax*) or hepatic schizonts are in categories TCP-3 and TCP-4, respectively. Drugs that block transmission to the mosquito by inhibiting gametocytes are TCP-5 compounds, and those that block transmission by targeting the insect vector are TCP-6 (endectocides). Two or more compounds are combined into clinical therapies with target product profile 1 (TPP-1) and TPP-2 (ref. 240). TPP-1 focuses on drugs for chemotherapeutic treatment of acute uncomplicated malaria in children or adults, using compounds with TCP-1; if possible, TCP-3, TCP-4 and TCP-5 compounds are added to reduce relapse, provide post-treatment prophylaxis and block transmission. TPP-2 focuses on prevention, in high-transmission areas or during epidemics, and gives protection via TCP-1 and, ideally, TCP-4 for prophylaxis and TCP-5 to clear gametocytaemia in asymptomatic individuals. Antimalarials approved for clinical use are listed for each TCP category.

The bar is high for treatments that will replace existing therapies. To facilitate the discovery and development of next-generation therapies, some criteria have been agreed on by physicians, scientists and patient advocates. The target candidate profile (TCP) defines a set of characteristics required for a chemical molecule to be used in malaria therapy. A description of the different antimalarial TCPs and the *Plasmodium* lifecycle are given in Fig. 1. The target product profile (TPP) relates to the minimum and ideal criteria for a combination product that is effective in the field and delivers value beyond that offered by the standards of care. There are two general categories of TPPs and multiple use cases. The first is a TPP for treatment (TPP-1), primarily aimed at new drug combinations for the management of uncomplicated malaria. However, there are important nuances: there is still a need for new medicines for patients with severe malaria, for whom oral medicines are not ideal, and for drugs that can be used to manage asymptomatic infections in the wider population, including killing dormant parasites in *P. vivax* malaria.

The second major target profile (TPP-2) includes chemoprevention (giving a full treatment dose to individuals in highly endemic areas to control transmission, as some individuals can be asymptomatic carriers) and prophylaxis (administering a drug to asymptomatic individuals at risk of infection). Given there is no fully protective vaccine, chemoprevention is currently the major approach to protect travellers or migrants, pregnant women (intermittent preventive treatment in pregnancy), children under 5 years, and children with other co-morbidities such as anaemia or sickle cell disease. However, with over 60% of the annual cases of malaria occurring during the rainy season, and with increasing threats of resistance to SP–amodiaquine, new drugs for SMC will be necessary. Determining appropriate alternatives to SP–amodiaquine requires the consideration of multiple factors, from drug resistance, safety and the pharmacokinetic–pharmacodynamic relationship, to availability and cost. Ideally, two or more highly potent antimalarial compounds with excellent safety profiles and different mechanisms of action, long terminal elimination half-lives, and compatible pharmacokinetic–pharmacodynamic profiles would allow for monthly SMC dosing. The development of safe and effective long-acting injectables is another strategy that could have a significant role in SMC, subject to being able to achieve wide population distribution and duration of protection.

Because malaria cases occur in tropical areas, it is important to confirm the chemical stability of the final product in high temperatures and humid conditions. Furthermore, since those affected by malaria are predominantly children under 5 years old, new molecules that are used in either TPP need to be well tolerated in children, which demands effective pharmacovigilance and analysis of population-specific genetic variations that can influence drug metabolism. Products also need to be formulated appropriately for children, which can include taste masking and dispersible formulations. Pregnant women are also at particular risk; therefore, reproductive toxicology testing during drug discovery and development is of utmost importance. Each year, 30 million pregnancies occur in malaria-endemic zones and access to pregnancy testing is often not available; hence, safety in early pregnancy is critical.

In malaria, as with many infectious diseases, the drug development process is more complicated because the optimal final medicine contains two or more active drugs in combination. Medicines for uncomplicated *P. falciparum* malaria must be fixed-dose drug combinations to prevent resistance and maximize compliance, but the process to discover these combinations needs to focus first on delivering a single compound as a candidate drug, and then on evaluating the best partners for combination therapy, considering the mechanism of action of each drug and their pharmacological matching.

Antimalarial drug discovery is also fundamentally different from that for other diseases prevalent in developed countries. The main burden of malaria in sub-Saharan Africa falls on the poorest populations; hence, medicines can be available for free in that region through public National Malaria Control Programmes and are procured through agencies like the Global Fund. The high cost of drug development, the limited commercial return, and the complex regulatory and reimbursement landscape led many pharmaceutical companies to exclude malaria and other tropical diseases from their portfolios at the end of the last century. In response to this unfortunate restriction, innovative drug discovery and development models have been achieved through product development partnerships, in which costs and risks are shared with partners, facilitating interactions (Box 1). One example of a product development partnership is the Medicines for Malaria Venture (MMV), which is funded through governments and philanthropic foundations to engage with both the public and private sectors. To ensure that a portfolio of antimalarial products is developed and made available to patients, MMV maintains multiple partnerships with drug discovery and parasitology experts to discover, develop and deliver new antimalarials. The role of MMV in delivery and access is critical: insights from maximizing the impact of the current medicines are important to set the goals for the next generation of treatments.

## Antimalarial drug resistance

*P. falciparum* resistance to antimalarial drugs has long been the scourge of global efforts to effectively treat malaria and reduce the disease burden^11^. With notable exceptions of the approved combinations AL (see below) and artesunate–pyronaridine, most antimalarial therapies have encountered resistant parasites in the field^10^. The early twentieth century saw reports of resistance to quinine, a drug credited with helping eliminate malaria from Europe after having been brought there from South America^12^. Resistance to chloroquine and SP, which swept across malaria-endemic regions starting in the 1950s, precipitated a major increase in malaria deaths later in the twentieth century until the 2000s, when ACTs were adopted globally as first-line treatment^13,14^. In addition, resistance has compromised the clinical efficacy of DHA–PPQ throughout most of the Greater Mekong Subregion (GMS) in Southeast Asia^15^. This, in turn, has led to the adoption of artesunate–mefloquine as one of the regional first-line therapies, despite this combination having previously encountered resistance^16^. The most widely used ACT (AL) remains clinically effective in the African continent. Importantly, ART partial resistance has been confirmed in Rwanda^17–20^. Another recent report showed that *P. falciparum* clinical isolates from Uganda had a decrease in susceptibility to lumefantrine and DHA as assessed experimentally in culture, raising more concerns of potential evolution towards a state of resistance^21^ and thereby increasing selective pressure on the partner drugs. The combination of pyronaridine and artesunate is minimally used, possibly because of higher costs, but remains an important alternative to AL.

### Drug resistance and ART

Considerable insights have been acquired into the mechanisms by which *P. falciparum* parasites have acquired resistance to antimalarial drugs. In the case of ART, mutations in *kelch13* (encoding K13) drive partial resistance by enabling a subset of ART-treated early ring-stage parasites to survive drug treatment^21–26^.

Clinically, this manifests as delayed parasite clearance in patients treated with an ART derivative or an ACT, with parasites persisting after 72 h. However, delayed clearance does not have a detrimental impact on the clinical and parasitic response rate by day 28, so long as the partner drug retains efficacy. In vitro, resistance is often defined as >1% of early ring-stage parasites surviving a 6-hour pulse of 700 nM DHA. In parasites at the asexual blood stage, K13 protein has been localized to sites on the plasma membrane, where haemoglobin is captured in vesicles that then traffic to the developing digestive vacuole. Mutations in the *kelch13* gene reduce its protein levels and decrease haemoglobin endocytosis^27,28^. The consequence is presumably lower levels of Fe^2+^–haeme, a product of haemoglobin degradation that activates ART by cleaving its endoperoxide bridge^29^. Reduced drug activation is thought to allow the survival of a subset of K13-mutant parasites. Mutant K13 has also been associated with multiple other cellular effects, including upregulation of the unfolded protein response, reduced proteotoxic stress, altered mitochondrial physiology, and extended ring-stage development coupled with faster progression through the more metabolically active trophozoite stages^30–35^. DHA-treated K13-mutant parasites also display increased susceptibility to the electron transport chain inhibitor atovaquone^33–35^. These findings lead to the intriguing possibility that mitochondria might sense initial cellular damage resulting from ART action and, in the presence of mutant K13, these organelles might help regulate entry into quiescence, with subsequent reemergence after this rapidly cleared drug drops to sub-inhibitory concentrations^35^.

The impact of mutant K13 on ART resistance and its spread in the parasite population is also dictated by the specific mutation and parasite genetic background. For example, despite not conferring the highest level of resistance in vitro, C580Y is the dominant mutation in the eastern GMS^36^, where it has spread in parasite sub-lineages that might potentially also harbour secondary facilitators such as proteins that either augment resistance or mitigate the impact of mutant K13 on normal parasite physiology. The C580Y mutation might also benefit parasite transmissibility, although this requires further research.

Gene editing studies have shown that African strains differ substantially in the degree to which K13 mutations impart resistance, with some strains showing little to no resistance in vitro^37,38^. Those studies also identified a detrimental impact of these mutations on rates of asexual blood-stage growth in *kelch13*-edited African strains that acquired ART partial resistance, suggesting that, in the field, these genetic backgrounds might not be competitive with wild-type strains. These issues of parasite genetic backgrounds and fitness might be key influencers of how quickly mutant K13-mediated ART resistance can emerge and spread in Africa, where there might be different human and parasite genetic variants relative to Asia, different patterns of drug use, and different levels of immunity. Other mediators of resistance have also been identified in vitro, including coronin, AP2μ, ubp1 and KIC-7 (refs. 28,39–41). Screening for variants of these genes, in addition to *kelch13*, could be informative in genomic surveillance efforts. Finally, it might be possible to find more genes involved in ART resistance. Recently, genome analysis on over 2,000 samples of *P. falciparum* isolates from 24 malaria-endemic settings in 15 African countries indicated shared genomic haplotypes, especially in drug resistance loci^42^. One example was on chromosome 12, where candidate resistance loci against ART derivatives were identified in samples collected in Ghana and Malawi.

ACTs only fail when both the ART derivative is compromised and the partner drug encounters resistance^43^. In the GMS, DHA–PPQ failed as a result of the local spread of parasites resistant to both drugs^15^. The acquisition of parasite resistance to PPQ appears to have begun with amplification of tandem genes encoding Plasmepsins 2 and 3 (refs. 44–46). These enzymes contribute to haemoglobin proteolysis and form a complex that facilitates haemozoin formation^47,48^. The amplification event appears to enable some parasites to withstand high concentrations of PPQ, a drug that acts, at least in part, by binding haeme and inhibiting its detoxification^49,50^. High-grade resistance to PPQ is mediated primarily by mutant forms of the chloroquine resistance transporter PfCRT, which in Southeast Asia typically occurs in parasites with multicopy Plasmepsins 2 and 3 and mutant K13 (refs. 51–53). These isoforms enable PfCRT to transport PPQ, presumably redirecting the drug away from its haeme target in the digestive vacuole^54^. Evidence suggests that multiple PfCRT variants initially arose, reducing over time to a select few that achieved both resistance to elevated drug concentrations and low to no fitness cost^44,51,52,55^. Many of these mutations also lead to a loss of chloroquine resistance and can increase parasite susceptibility to the related drug amodiaquine^51,52,56^. Modelling studies predict that these opposing effects on PPQ and chloroquine susceptibility could be leveraged into multidrug treatment regimens that exert opposing selective pressures on PfCRT and suppress the emergence of resistance^56^. Indeed, the concept of combining drugs with opposing selective pressures on PfCRT, and the related digestive vacuole-resident drug transporter PfMDR1, is part of the rationale for testing the triple ACTs DHA–PPQ plus mefloquine and AL plus amodiaquine, both of which proved highly effective in areas where resistance is reported against ART and a second drug^57,58^. Studies also support the use of multiple first-line therapies, such as AL and AS–amodiaquine, that exert opposing selective pressures on PfCRT and PfMDR1, with the intent of suppressing the emergence and spread of multidrug-resistant parasites^59^. DHA–PPQ is also being considered as a replacement for SP–amodiaquine in chemoprevention measures in Africa, particularly considering the widespread prevalence of SP-resistant parasites^60,61^.

Many of these resistance studies are focused on compounds that are in clinical use. There is ongoing debate about how much resistance risk can be tolerated for compounds in the development pipeline. For example, in a phase II dosing study of cipargamin (KAE609) monotherapy, approximately two-thirds of patients that recrudesced (34 of 133 patients) carried a concerning mutation (G358S) in PfATP4 (refs. 62,63). However, in this study, the drug was administered as a monotherapy and different dosing regimens were being tested. To try to model this, stakeholders have defined an in vitro minimum inoculum of resistance (MIR) plus a resistance threshold (fold IC_50_ shift) as a way to quantify risk^64^, although emerging data suggests that parasite fitness also needs to be considered. Measuring the MIR is a time-consuming activity, but early studies must be conducted to remove the most resistance-prone chemical scaffolds before initiating chemistry optimization phases. To date, all compounds tested that inhibit a defined parasite target selectively could generate some level of resistance in vitro within 60 days. Novel antimalarial therapies are expected to be developed as combination therapies to minimize the risk of resistance.

## Approaches to antimalarial drug discovery

The spread of *P. falciparum* resistance to current first-line antimalarials highlights the need to rapidly develop new drugs with novel modes of action and resistance. Historically, this has followed several strategies, ranging from the isolation and modification of natural products to the screening of compound libraries and the design of inhibitors for known targets^65^. Although natural products have been successfully used as frontline medicines, natural product-driven malaria drug discovery is beset by a number of challenges^66^. These challenges range from reliable access and supply or resupply to variable composition of the source (due to environmental factors) and practical challenges associated with complex mixtures following bioassay-guided fractionation. In addition, high-throughput screens (HTS) of libraries of natural products and extracts have not yet identified any hits of sufficient quality. Although it might be possible to synthesize new versions of established natural products (such as ART), the difficulty in improving the field resistance profiles makes continued work on these scaffold families less attractive. Thus, almost all new antimalarial drug classes today are discovered via a combination of phenotypic and target-based screening of small synthetic molecules.

### Phenotypic screening

In a typical phenotypic screening approach to identify new starting points for antimalarial drug discovery, large chemical libraries of synthetic small molecules are profiled against whole-cell parasites cultured at desired lifecycle stages to determine their in vitro activity. The target-free principle of this method allows unbiased screening against all druggable targets, contingent on the presence of active molecules in the tested library of compounds. Examples of successful HTS against *P. falciparum* asexual blood-stage parasites have been reported^67–70^.

This approach has the advantage of accounting for properties of the compound, such as its cell permeability and on-target interactions, and provides the greatest potential for finding potent compounds with novel modes of action^71^. Indeed, many antimalarials in the pipeline were discovered from phenotypic screening, including ganaplacide (KAF156), cipargamin (KAE609), cabamiquine (M5717, DDD107498, MMV643121), and ZY-19489. More recently, phenotypic screens have been performed on liver and gametocyte stages^72,73^. In addition, asexual blood stage-focused screens have been modified with extended incubation time to identify slower-acting inhibitors that would have been missed in shorter incubation assays^74^.

Unbiased target-free phenotypic campaigns pose a subsequent challenge because, without knowledge of the target, it can be difficult to rationally improve potency^75^. One way to address this is by screening a focused library of inhibitors that act against known mammalian target classes, which might therefore also inhibit their *Plasmodium* ortholog. A series of potent amino-amide boronates, which selectively inhibit the *P. falciparum* 20S proteasome, was recently identified and rationally optimized with this strategy^76^ as was a series of aspartic protease inhibitors that were demonstrated, via resistance selection, to act by targeting the *Plasmodium* aspartic proteases Plasmepsins IX and X (PMIX and PMX)^77^.

### Target-based and virtual pharmacophore approaches

The target-based approach is complementary to phenotypic screening and provides the opportunity to develop new biochemical assays for a promising new target. It also allows compounds to be rationally designed with better potency and selectivity against the target, including the use of methods such as screening DNA-encoded libraries, fragment-based and structure-based hit generation, and virtual screening^78^, in which compound structures are computationally docked into protein structures.

Target-based biochemical screens have been executed against a variety of malaria targets and have yielded improved molecules with both phenotypic and biochemical activity. A chemically and clinically validated target in *P. falciparum* is dihydrofolate reductase–thymidylate synthase (DHFR-TS), against which the drug candidate P218 was optimized using a structure-guided enzymatic inhibition assay^79^. Hits from target-based screens are often weak and require further optimization to achieve the desired phenotypic profile against whole-cell parasites. In the first HTS for inhibitors of the mitochondrial enzyme dihydroorotate dehydrogenase (DHODH), hits lacked whole-cell antiplasmodial activity^80^ but, after selection of alternative series and chemical modifications to optimize DHODH binding, the clinical candidate DSM265 was discovered^81^. DSM265 delivered a successful proof of concept in a phase IIa monotherapy study. Interestingly, several patients recrudesced who were harbouring parasites resistant to the drug with mutations in DHODH^82^. Anecdotally, there appears to be a correlation between resistance risk and the use of target-based drug discovery, as exemplified by DSM265. Measuring resistance risk (such as by lengthy MIR studies) is challenging and, therefore, it cannot yet be used frequently during the medicinal chemistry optimization cycle in drug discovery. This does not necessarily mean that resistance risk cannot be thoughtfully minimized in the future.

An additional problem with target-based screens is that they can be expensive, limiting the size of a chemical library that can be screened. In contrast, virtual screens do not require expensive reagents and can be more cost-effective for an initial triage. Predicted active compounds need to be acquired and substantial downstream work is necessary to confirm actual binding to the target and functional inhibition of the parasites. Virtual pharmacophore screens have recently been employed in the search for new chemical starting points active against *Plasmodium* targets, including the DNA minor groove^83^, DHODH^84^, falcipain 2 (ref. 85), β-haematin formation^86,87^ and metalloaminopeptidases^88^. However, this strategy has not yet yielded novel compounds with potent cell-based activity. Pharmacophore models, based on either the protein target or a ligand binder, are nonetheless important tools in the optimization of compounds identified through phenotypic and biochemical screens. Machine-learning approaches can be also applied without the pre-selection of a target. Regenerative modelling based on neural networks (JAEGER) was used to search for novel chemical matter with desired bioactivity and to identify phosphatidylinositol 4-kinase (PI4K) inhibitors with low nanomolar activity in a biochemical assay as well as against *Plasmodium* species in a parasite proliferation assay^89^.

### Approaches to finding new druggable targets

Target-based drug discovery relies on having well-validated targets. The ideal target (whether protein or RNA) should be essential to parasite life and also chemically and clinically validated. A wide variety of chemically validated targets is desirable to allow a broader chemical space to be explored. In addition, the target should be novel to minimize the possibility of pre-existing resistance. Clinically validated malaria targets (Table 1) include the targets of licensed drugs cytochrome B (CytB), dihydropteroate synthase (DHPS), DHFR-TS and haeme as well as newer targets such as ATPase4 (ATP4), elongation factor 2 (eEF2), prolyl tRNA synthetase (PRS) and PI4K. Interestingly, inhibition of many of these targets leads to rapid parasite death, which translates into good patient efficacy provided the parasite is sensitive and there is sufficient compound exposure. There are also several antimalarial targets with in vivo validation that have not yet been clinically tested (Table 2) such as phenylalanine tRNA synthetase (FRS) and acetyl-CoA synthetase (AcAS). Antimalarial targets in early stages of development with in vitro validation include cytoplasmic isoleucine–tRNA ligase (cIRS) and farnesyl pyrophosphate–geranylgeranyl diphosphate synthase (FPPS–GGPPS) (Table 3).

Many target identification discoveries involved the Malaria Drug Accelerator (MalDA), a consortium of 18 different groups^90^. The MalDA consortium has classified and prioritized targets based on their intrinsic properties as well as the characteristics of the compounds that inhibit them. For example, the best targets should be inhibited by tool compounds that are fast-killing in a parasite reduction rate assay, that do not readily develop resistance in MIR assays, and that act across the lifecycle. The tool compounds should also show similar potency across a variety of *P. falciparum* lines that bear mutations in multidrug resistance genes such as *pfcrt* and *pfmdr1*. The rationale for these requirements is that, since optimized compounds need to have these criteria to progress, tool compounds should as well. On the other hand, some of these parameters are clearly compound specific and one tool compound for a target might have a resistance risk and another might not, depending on the binding mode. Target identification strategies can be classified into those where tool compounds are available (compound dependent) and those where tool compounds are not available (compound independent) (Table 4). These methods each have advantages and drawbacks and can be applied alone or in combination to discover druggable targets.

#### Compound-dependent approaches.

Phenotypic screening campaigns performed over the past decade have created large collections of tool compounds that have been used to identify targets. The most successful compound-dependent method is in vitro evolution and whole-genome analysis, which has led to the discovery of various targets, including ATP4, eEF2, PI4K, and AcAS^91,92^ and most tRNA synthetase inhibitors (KRS, cIRS, PRS and FRS). The method entails culture- or animal model-based selection of parasites to obtain genotypes resistant to the compound of interest. This is followed by comprehensive whole-genome analysis of resistant clones to pinpoint the locations of the mutations responsible for resistance and therefore a potential target gene. Despite the success of this method, it can be difficult to evolve resistance to a compound, and resistance can occur due to non-specific mutations in pleiotropic resistance determinants, such as the transporters PfMDR1 or PfCRT, rather than due to a mutation in the target gene. Other compound-dependent methods include proteomic approaches such as affinity chromatography (which led to the identification of PI4K^93^ and PKG^94^ as targets) and the cellular thermal shift assay^95^. Furthermore, overexpression libraries, in which parasites are transfected with a library containing genes, possibly on cosmids, in order to test for a gain of resistance to compounds of interest, have been successfully applied in other parasites^96,97^ and hold promise for finding novel *Plasmodium* targets.

In silico approaches, including structural docking or compound similarity searches to inhibitors with known targets or mechanisms of action, have not been used as frequently as the other methods. However, the mitochondrial target of atovaquone, CytB, was likely discovered based on the similarity of atovaquone to the naphthoquinone inhibitors, which have mitochondrial targets.

Finally, there are compound profiling approaches, including metabolomics or transcriptomics, in which a pattern is matched to the pattern of a known inhibitor^98^; these methods have been successful, especially for certain classes of metabolic inhibitors such as CytB, DHODH and DHFR inhibitors^67^. In all cases, targets can be further validated using conditional knockdown methods^99^ as well as by protein overexpression and demonstration of direct binding using either biochemical assays or biophysical methods such as surface plasmon resonance and crystallography. In many cases, different deconvolution methods can give the same result. During the lead optimization phase, MMV390048 underwent mode of action and resistance investigations that confirmed PI4K as the primary target, based on chemoproteomics via pull-down experiments as well as in vitro evolution studies. In many cases, targets that were discovered and validated using genomic approaches (such as DHODH) were rediscovered using target deconvolution methods.

A protein target class that has appeared repeatedly and unexpectedly with in vitro evolution methods is the tRNA synthetase group of enzymes. They catalyze the charging of tRNAs with amino acids in an ATP-dependent reaction and are essential for protein synthesis. For *P. falciparum*, the 36 predicted tRNA synthetases (which are abbreviated using the amino acid code plus R for tRNA and S for synthetase) can be targeted to the apicoplast, mitochondria and/or cytoplasm. Importantly, many tRNA synthetase inhibitors appear to be active against liver-stage parasites, making them ideal candidates for both TCP-1 and TCP-4 class inhibitors. Target deconvolution with the natural product cladosporin led the way in identifying KRS as a target^100^, and the development of specific inhibitors is under way^101^, including against FRS^102^, PRS^103^, leucine–tRNA ligase^104^, YRS^105^ and, most recently, IRS^106^. These targets have been well validated and a number of them are now structurally characterized^105,107,108^. The tRNA synthetase class also appears to be an important target class in other pathogens such as *Mycobacterium tuberculosis*^109^, *Leishmania* species^110,111^ and *T. gondii*^112,113^, and tool compounds show specificity despite orthologs existing in humans. The high level of species selectivity might be achieved because, in humans, tRNA synthetases are part of multiprotein complexes and the enzyme active site is less accessible and thus less druggable.

In vitro evolution can also be used to establish on-target activity for compounds discovered in biochemical screens such as inhibitors of cyclin-dependent-like kinase 3 (CLK3)^114^ or purine nucleoside phosphorylase (PNP)^115^. The approach has confirmed that compounds predicted to be proteasome inhibitors do indeed target this protein complex^76,116–122^. Interestingly, the proteasome is another example of a target that is important in other pathogens and, as with *Plasmodium*, evolution has been used to establish on-target activity: whole-genome RNA interference screening in *Trypanosoma brucei* and resistance generation in *Leishmania donovani* confirmed the proteasome to be the target of the GSK3494245 scaffold, and one compound from the series is now a preclinical candidate to treat visceral leishmaniasis^123^. Likewise, in vitro evolution was used to confirm the proteasome as the target of GNF6702, a compound active against leishmaniasis, Chagas disease and sleeping sickness^124^. It was also used, along with multiomic approaches, to show that the proteasome is the target of arylsulfonamides in *Trypanosoma cruzi*, the causative agent of Chagas disease^125^.

#### Compound-independent approaches.

Compound-independent approaches typically involve genome sequence analyses, such as searches for targets that are known to be druggable in other species or identifying protein families that are present in *Plasmodium* parasites but missing in humans. There was substantial activity in this area after completion of the *P. falciparum* genome sequence, with particular attention given to proteins associated with the apicoplast, a relic bacterial organelle missing in humans. It was first noted that fatty acids are synthesized in the apicoplast using a type II pathway^126^. Later, it was shown that the sole essential function for this organelle in asexual blood-stage parasites is the production of isoprenoids and that the provision of isoprenyl pyrophosphate enabled apicoplast-deficient parasites to survive^127^. Targeting the prokaryotic-like apicoplast translation machinery with antibiotics kills parasites via a delayed death mechanism that only occurs in the second generation of parasite development^128^. This is less desirable because symptoms would not resolve quickly and could potentially increase the risk of death in cases of severe malaria. These data also highlight the importance of not relying on genome sequences to find asexual blood-stage targets as many parasite gene products could be expressed at other clinically irrelevant stages of the parasite lifecycle such as in the mosquito stages. Nevertheless, apicoplast-targeting drugs could have some value, especially in the context of prophylaxis, where individuals are asymptomatic and the rate of parasite clearance is less important, as well as in combination therapy. Screens have been devised to find starting points for future medicinal chemistry efforts^74^, and genes that are essential for apicoplast function are being identified^129^.

Genome-based target identification approaches are more reliable when they are combined with techniques to determine the essentiality of genes during asexual blood-stage development. A clever strategy involved identifying *P. falciparum* genes whose disruption with transposon mutagenesis^130^ led to a fitness disadvantage. Likewise, a genome-wide knockout library of rodent *Plasmodium berghei* was used to identify essential blood-stage genes^131^ as well as genes and metabolic processes essential for liver-stage development^132^. These approaches seem to have accurately identified genes that are critical for parasite survival, and several targets have emerged from genomic methods, including the histone acetyltransferase GCN5 and the clinically validated DHODH enzyme^82^. However, a target identified as genetically essential by genomic methods might not be druggable.

## The antimalarial drug development pipeline

**Fig. 2 |:**
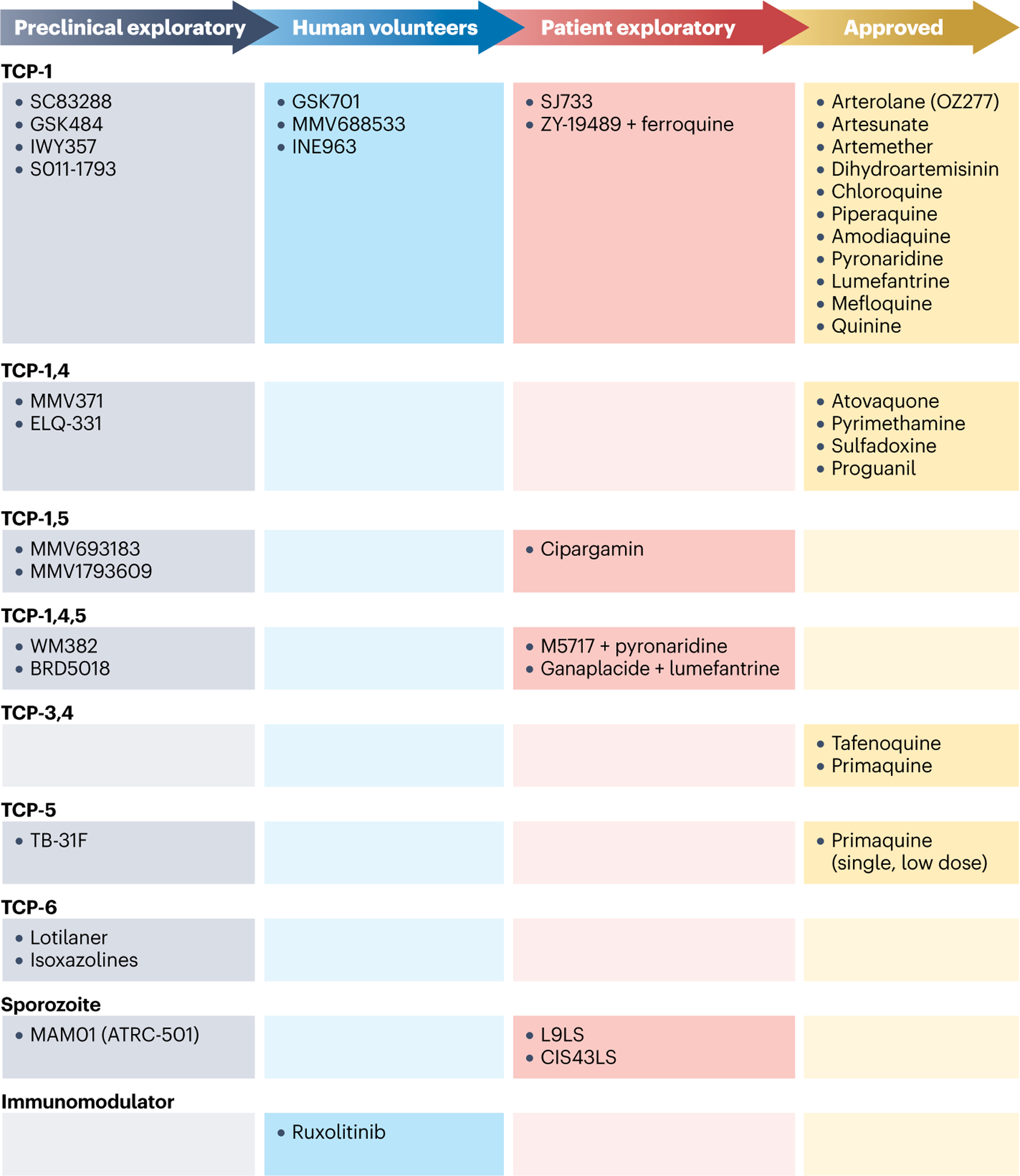
Antimalarial drug discovery and development pipeline. Drug candidates in preclinical exploratory phase, human volunteer phase and patient exploratory phase as well as approved drugs are listed. They are grouped in rows according to their target candidate profile (TCP), which represents the stage of disease being targeted. Monoclonal antibodies MAM01 (ATRC-501), L9LS and CIS43LS block hepatocyte infection from sporozoites. Ruxolitinib is an immunomodulator and is being tested in combination with antimalarial therapy to modulate the long-term host immune response.

The list of antimalarial compounds in development, as of 2022, is shown in Fig. 2, highlighting their TCPs; the list is also regularly updated on the MMV website^133^. The drugs that are approved and in use against malaria are also provided in Fig. 2 but will not be discussed further here.

### Patient exploratory phase

In the patient exploratory phase, compounds are administered to patients with malaria, representing the most advanced compound candidates in the last stages before approval. These studies have the potential to reveal whether medicines remain effective against field parasites, which are typically genetically very diverse. Drug candidates to treat malaria that were undergoing clinical exploratory and confirmatory studies between 2016 and 2021 were recently reviewed^134^. In addition to small molecules, the NIH is testing two monoclonal antibodies (CIS43LS and L9LS) in proof-of-concept and clinical prophylactic studies. These antibodies were designed to bind and neutralize circumsporozoite protein, a major parasite surface protein essential for pre-erythrocytic stage infection. Initial clinical results with L9LS demonstrated efficacy in protecting recipients from malaria when sporozoites were administered intravenously or subcutaneously, without evident safety concerns^135^. For more information about monoclonal antibodies and alternative approaches to malaria therapy see Box 2.

Novel small molecules, all of which are specific for malaria, that have progressed to studies in patients with malaria include ZY-19489 (also known as AZ13721412 and MMV674253), which was optimized following identification of the original triaminopyrimidine hit during HTS^136^ and licensed to Zydus. ZY-19489 is a fast-killing asexual, blood-stage-acting (TCP-1) compound with a low propensity to select for resistance (deemed ‘irresistible’). Promising results on safety, pharmacokinetics and antimalarial activity in a volunteer infection study (VIS) support its further development as an antimalarial therapy^137^. VIS enables drug potency to be assessed with asexual blood-stage or liver-stage *P. falciparum* parasites in volunteers^138^. This carefully controlled protocol provides valuable information on the concentration–response profile of the drug in humans with *P. falciparum* infection, thereby facilitating dose selection for subsequent clinical studies. Ferroquine, a fast-killing and long half-life 4-aminoquinoline drug that incorporates a ferrocenyl ring in its alkyl amino side chain^139^, has also been licensed to Zydus. Modelling of a ZY-19489 and ferroquine combination suggests that single-dose efficacy in children might be achievable, which would combine two irresistible compounds with long durations of action. This is a potential product that could be used in the event of emerging resistance to the mainstay ACTs such as AL.

The candidate drug cabamiquine, licensed to Merck KGaA^140^, was optimized from an interesting 4-aminoquinoline hit discovered in a phenotypic screen of a small library of kinase inhibitors. Its molecular target was identified as eEF2 by analyzing resistance mutations, supported by the observation that the compound inhibits protein synthesis^141^. Cabamiquine has potent activity on parasite asexual liver and blood stages and sexual stages of the parasite, with excellent causal prophylaxis and transmission-blocking properties (TCP-1, TCP-4 and TCP-5). Healthy volunteer studies and VIS were conducted to confirm the efficacy and safety of the compound. Given that cabamiquine rapidly selects for resistance, pyronaridine was selected as an irresistible partner drug^142,143^.

Cipargamin was discovered by scientists within the global NGBS consortium (Novartis Institute for Tropical Disease, Genomics Institute of the Novartis Research Foundation, Biomedical Primate Research Centre, and Swiss Tropical and Public Health Institute). Resistance is mediated by mutations in the gene encoding PfATP4 (a protein on the parasite membrane that modulates sodium-proton exchange), suggesting that this is the target of the small molecule^144–147,62,63^. Treatment with cipargamin results in asexual blood-stage parasite rigidification and rapid clearance in vivo. The compound is also potent in blocking transmission (TCP-1 and TCP-5). The efficacy and safety of cipargamin have been confirmed in healthy volunteers and patients, where it delivers a parasite clearance rate even more rapid than that of ART. A reformulated version as an injection for use in severe malaria is being explored. Given the known risk of resistance to cipargamin^148^, a combination will be required to protect its longevity and ensure patient efficacy.

The most advanced new compound is ganaplacide, an imidazolopiperazine also known as KAF156, which was discovered within the NGBS consortium. Resistance to this compound as well as to the closely related compound GNF179 can be mediated by mutations in the *P. falciparum* cyclic amine resistance locus (CARL; PF3D7_0321900) or via mutations or stop codons in the endoplasmic reticulum-localized acetyl-CoA transporter (ACT) or UDP-galactose transporter (UGT)^149^. PfCARL encodes a relatively uncharacterized transmembrane protein expressed in the *cis*-Golgi compartment^150^ that appears to be associated with resistance to a variety of compounds^151^, and ACT is non-essential in vitro. Therefore, the specific target of ganaplacide is unidentified, although all data suggest that it might have a role in protein processing or secretion. Ganaplacide is fast-killing and potent in the asexual liver and blood stages as well as in the sexual stages of *P. falciparum* (TCP-1, TCP-4 and TCP-5), thus demonstrating the potential for TPP-1 treatment with the additional benefits of post-treatment prophylaxis and transmission-blocking activity. Its potency against asexual blood and liver stages and favourable pharmacokinetic and safety profiles have been confirmed in healthy volunteer and patient studies (clinical phases I and II)^152^.

Ganaplacide is combined with lumefantrine, a partner drug used in ACTs for which Novartis now has a new formulation optimized for daily dosing with improved solubility and oral bioavailability. This formulation should help reduce the risk of resistance and maintain efficacy^153^. Novartis announced positive results from an efficacy clinical study with ganaplacide plus the optimized lumefantrine formulation, and a decision to move forward to phase III with the combination has been announced^154^. If this combination continues to show efficacy in larger cohorts of patients, then it has the potential to be the first non-ACT treatment since Malarone (atovaquone–proguanil) was launched in 2000 and would be an option in the event of ART resistance emerging.

### Human volunteer phase

Compounds being studied in phase I clinical trials include a reformulation of atovaquone–proguanil named atoguanil^155^ and two new chemical entities: MMV688533 (ref. 156) and INE963 (ref. 157). The TCP-1 candidate drug MMV688533 was discovered in 2017 using an orthology-based screening strategy, where the chemical library was based on compounds active against druggable targets in other species for which an ortholog exists in *P. falciparum*. Screening yielded an impressively high hit rate. However, one key hit was inactive upon resynthesis, and chemists identified a minor impurity that contained all the activity. Optimization of the identified active molecule yielded MMV688533, a fast-killing compound with a long predicted human half-life, to which resistance could not be achieved in laboratory conditions^156^. Although its mode of action remains unknown, selection studies identified two low-grade resistance mediators to MMV688533: aspartate-glutamate carrier isoform 1 (PfACG1) and EH domain-containing protein (PfEHD). Both determinants are implicated in intracellular trafficking, lipid utilization and endocytosis^156^. MMV688533 has acceptable human tolerability, is completing clinical phase I in a VIS, and will contribute to the pool of next-generation antimalarials to be assessed in phase II treatment combinations after cabamiquine–pyronaridine and ZY-19489–ferroquine.

INE963 was recently discovered by the Novartis Institute for Tropical Disease after an initial phenotypic screen that identified active imidazothiazoles. Optimization delivered this TCP-1 candidate, which is a fast-killing compound acting at the asexual blood stage with a low propensity to select for resistance. This compound recently entered phase I clinical trials^157^ and will also be assessed in phase II trials of treatment combinations.

The transition between human proof of concept with laboratory strains and studies with patients has been a point where investigational interventions might fail, notably in some phase II studies with malaria vaccine candidates where infections with heterologous parasites occur^158^. As an example of unrelated, inevitable attrition associated with drug development, the DHFR inhibitor P218 (TCP-1 and TCP-4)^159^ progressed to clinical studies and a liver-stage VIS, but results showed a short half-life in humans and thus development was halted^79^.

### Preclinical exploratory phase

During development of a new chemical entity, the first phase of chemistry is called hit-to-lead, with the culmination being an ‘early lead’. The second phase is lead optimization, with the milestone being a ‘late lead’. This late lead is then profiled further (safety and other studies) to assess whether the preclinical candidate criteria have been met. The criteria to classify a molecule as a hit and as a lead have been previously discussed^160^. Compounds that reach the preclinical exploratory phase have been extensively optimized by medicinal chemistry and meet the criteria described in Table 5.

There are 11 candidate small molecules and one antibody being investigated in the preclinical exploratory phase to determine whether they have sufficient predicted human pharmacokinetics and efficacy and can be safely dosed in humans.

Many of the compounds at this stage were discovered in phenotypic screens and their mechanisms of action have been determined or confirmed using a combination of methods (Table 4). MMV183 (also known as MMV693183) is an antimetabolite that inhibits AcAS and has potent transmission-blocking and asexual blood-stage activity (TCP-1 and TCP-5). This compound is derived from the natural product pantothenic acid (vitamin B5)^161^. GSK701 (also known as MMV1582367), whose precursor was discovered by GSK in a phenotypic screen, is a novel inhibitor of the acyl-CoA synthetases ACS10 and ACS11 in clinical phase I, with a TCP-1 profile. Merck, in collaboration with WEHI, identified the compound WM382 as an inhibitor of PMIX and PMX^77^, and BRD5018 was discovered as an FRS inhibitor by a collaboration between Eisai and the Broad Institute. Both WM382 and BRD5018 have TCP-1, TCP-4 and TCP-5 profiles. PMIX and PMX are involved in egress and invasion and are novel targets.

A mechanism of action, though desirable, is not necessary for a compound to advance. SC83288 is a novel injectable candidate for severe malaria with an unknown mode of action^162^. GSK484 (also known as MMV1793192) was discovered from a phenotypic screen and displays a fast-killing, irresistible TCP-1 profile^163^. IWY357 was discovered as a TCP-1 compound by Novartis and is fast-killing with a low propensity to select for resistance^164^. Structures and mechanisms of action have not been published for the latter two compounds.

Some of the discovery-stage compounds include chemical improvements to clinically validated compounds: MMV371 (also known as mCBE161) is an atovaquone prodrug for injectable prophylaxis targeting the parasite CytB Q_o_ site (TCP-1 and TCP-4) delivered by Calibr^165^. ELQ-331 (also known as MMV1579167) is an oral prodrug of the TCP-1 and TCP-4 compound ELQ-300 (ref. 166). Like MMV371, ELQ series compounds are inhibitors of CytB but interact with the Q_i_ site^167^. S011–1793 was discovered in India and is a next-generation 4-aminoquinoline that overcomes chloroquine and PPQ resistance (TCP-1)^168^. MMV1793609 (ref. 169) is a PfATP4 inhibitor with a predicted long human half-life and efficacy at low doses (TCP-1 and TCP-5) and was discovered in 2022 as a backup to the PfATP4 inhibitor SJ733 (ref. 170).

### Discovery phase

Globally, the antimalarial discovery phase portfolio currently comprises 30 chemical series that have been deemed sufficiently promising to undergo optimization or profiling with assistance from MMV. There are too many compounds to be discussed here and, in some cases, structures have not been made public. Although some compounds have unknown modes of action, known targets include some of those previously mentioned: AcAS, GPI-anchored wall transfer protein 1 (GWT1), KRS, PRS, YRS, proteasome, phosphodiesterase, DHODH, ATP4, haeme, CytB and PI4K. Target-based screens against priority targets, such as GCN5, FRS, CLK3 and AcAS, are anticipated to deliver new chemical series to the portfolio in addition to phenotypic screens on asexual blood and hypnozoite stages, in particular carried out by MalDA and MMV^90^. As described above, prioritizing targets is based on having tool compounds with a low rate of resistance generation (MIR) and a relatively low level of resistance, a fast speed of kill, target novelty, and availability of assays and protein structures.

### Deprioritized compounds

The distribution of active projects is always changing. In the last 5 years, several novel candidates have been evaluated in patient exploratory studies (clinical phase II) but are unlikely to progress further. For example, progression of the parasite DHODH inhibitor DSM265 stalled because of an accumulation of risks, including resistance, non-clinical toxicity and the complexity of the formulation^82,171^. Additionally, the PI4K inhibitor MMV390048 (ref. 93), with TCP-1, TCP-4 and TCP-5, was halted on the basis of non-clinical toxicology^172^. MMV390048 is notable as the first candidate drug discovered and developed entirely in Africa, the first antimalarial kinase inhibitor tested in humans, and the first antimalarial to have a phase I trial performed in Africa. Thus, despite both DSM265 and MMV390048 demonstrating excellent human pharmacokinetics and good tolerability in patients as well as requiring low to medium doses in VIS, there were other factors that resulted in attrition, emphasizing the difficulty of drug development. Another instructive example of the challenges of drug discovery is that of ozonide compounds. First, OZ277 (arterolane) showed sub-optimum exposure in a phase IIa clinical study and its development was halted. Revisiting the series with further optimization, the compound OZ439 (artefenomel) showed improved human pharmacokinetics^173^, but had formulation challenges leading to variable exposure in combination studies and was deprioritized^174^. Ranbaxy Laboratories in India pursued the development of arterolane and launched it as a combination product with PPQ as a 3-day treatment for *P. falciparum* malaria (Synriam).

Since 2005, there have been 19 clinical candidate drugs halted by MMV out of 34 candidate drugs delivered — a high number but an overall lower attrition ratio compared with the pharmaceutical industry across all indications. However, there have been invaluable lessons from these failures in terms of human dose prediction, resistance susceptibility and translatability of non-clinical assays.

### New products

In addition to new chemical entities, improved formulations can address a critical need. Potential products in phase III human clinical trials or regulatory review include new formulations of existing products for particularly vulnerable populations (such as infants) as well as the triple ACT AL–amodiaquine to prevent resistance. Some of the products in late-stage testing or approved include ACTs for uncomplicated malaria, intravenous or rectal artesunate for severe malaria, tafenoquine for radical cure, and SP–amodiaquine for SMC.

## Future challenges and perspectives

The bar for antimalarial compounds that are likely to advance into human trials has risen steadily. An ideal compound protects against malaria, kills gametocytes, is safe and well tolerated, can be used in pregnant women and children, cures in a single dose, and is inexpensive and easy to administer. Furthermore, it should also have a good resistance profile, be active against most circulating drug-resistant strains and be difficult to raise resistance to in laboratory-based selection studies. Much more needs to be learned. For example, most resistance mechanisms that are studied in the laboratory involve a single genetic change but, in field isolates, multiple mutations in addition to the primary mediator might be involved to enable successful dissemination of the resistant parasites. It is also important to include recently adapted parasite strains that have likely been exposed to ACTs and thus differ from substantially older parasite strains that were exposed to chloroquine. More information is needed to understand how much the genetic background affects the likelihood of drug resistance emerging. Experiments in parasites are often slow and take months to complete, limiting the number of compounds that can be examined. To understand the mutability of a target, it can be more practical to study it in a model system when such an ortholog exists. For example, using a system in which *P. falciparum* DHFR-TS was expressed in *Saccharomyces cerevisiae* harbouring an error-prone DNA polymerase, researchers were able to rapidly create and explore the functional consequences of many variants^175^. Targets conserved across species include KRS, which interacts with the natural pro duct cladosporin that kills both *S. cerevisiae* and *P. falciparum* in the nanomolar range^100^.

Delivery systems and pharmacokinetics also remain a challenge. For example, delivering chemopreventive drugs with month-long efficacy durations without the need for repeated administration is extremely challenging. Additionally, although long-acting injectable strategies have had a major impact in areas outside malaria^176,177^, there are challenges associated with achieving acceptable volumes and duration of action as well as the need to confirm safety given the difficulty in removing a drug once injected. The human VIS model^138^ can be used to test for both prophylaxis and treatment, which will enable characterization of pharmacodynamic potential early in the clinical development phase.

Another concern remains for radical cure drugs, for which there have been few advances (Box 3) despite decades of effort. While malaria in the GMS and elsewhere in Southeast Asia is in decline, *P. vivax* cases appear to be increasing in sub-Saharan Africa. Even though *P. vivax* cases represent approximately 2% of those for *P. falciparum* malaria^4^, they can enter a dormant phase in the liver (hypnozoites), and more work is required to understand the interactions between liver-stage parasites and their host hepatocytes.

Significant progress has been achieved in the past 15 years on novel approaches towards malaria control and elimination, including the identification of novel druggable targets, a robust development pipeline of promising candidate drugs, and several new treatments. The first malaria vaccine (Mosquirix) was approved. Collaborative efforts across research organizations, the pharmaceutical industry, public sectors and philanthropic agencies, structured under a product development partnership model, have been instrumental in this success. Despite this measurable progress, no novel chemical entities have been licensed to treat *P. falciparum* malaria during this period, and the annual number of malaria cases persists, highlighting the vital need for new medicines. COVID-19 has provided a blueprint for accelerated research and development in the form of innovation and significant public and private sector investment, which should inspire the malaria community to reconsider drug development approaches and required levels of engagement. Innovations in scientific research and intensified and coordinated efforts from scientists, industrial partners, funders, stakeholders, public health institutions and health-care systems across the globe hold the promise that, in the foreseeable future, this disease can be largely vanquished.

## Figures and Tables

**Table 1 |:** Antimalarial targets with clinical validation

Target (systematic name)	Clinical stage	Mechanism of action	Noteworthy inhibitors	Refs.
Cytochrome B; CytB (PF3D7_MIT02300)	Approved (atovaquone)	Subunit of cytochrome *bc*_1_ complex; has a critical role in the electron transport chain and ATP production	Atovaquone (Q_o_ site); ELQ-331^a^ (Qi site)	178,179
Haeme	Approved	Haemoglobin degradation is critical to malaria parasite blood-stage growth	Chloroquine, amodiaquine, piperaquine	180
Dihydrofolate reductase–thymidylate synthase; DHFR-TS (PF3D7_0417200)	Approved (pyrimethamine, cycloguanil)	A dual-function enzyme that creates precursors for DNA synthesis	Pyrimethamine, cycloguanil, P218^a^ (DHFR site)	181
Dihydropteroate synthase; DHPS (PF3D7_0810800)	Approved	Catalyzes the last step in folate acid biosynthesis	Sulfadoxine	182
Deoxy-d-xylulose 5-phosphate reductoisomerase; DXR (PF3D7_1467300)	Approved	Catalyzes the non-mevalonate pathway	Fosmidomycin	183
Prolyl tRNA synthetase; PRS (PF3D7_0925300)	Approved	Essential for protein biosynthesis in all replicating parasite stages	Halofuginone	184–186
70S ribosome	Approved	Essential for protein synthesis	Macrolide antibiotics (such as azithromycin)	187
Dihydroorotate dehydrogenase; DHODH (PF3D7_0603300)	Phase II trials	Generates pyrimidine precursors for DNA synthesis	DSM265	81,188–191
PfATPase4; ATP4 (PF3D7_1211900)	Phase II trials	Maintains sodium homeostasis in parasite blood stages	SJ733; cipargamin	62,170, 192–194
Phosphatidylinositol 4-kinase; PI4K (PF3D7_0509800)	Phase II trials	Predicted lipid kinases with an essential role in trafficking	KDU691; MMV390048	93,195–198
Elongation factor 2; eEF2 (PF3D7_1451100)	Phase II trials	ATP-dependent enzyme with a critical role in protein biosynthesis	Cabamiquine (M5717)	142,143,199
Multidrug resistance protein 1; MDR1 (PF3D7_0523000)	Not available	Digestive vacuole membrane-localized ABC transporter	Multiple, including ACT-451840	200

a Compounds in clinical studies.

**Table 2 |:** Antimalarial targets with in vivo validation

Target (systematic name)	Mechanism of action	Noteworthy inhibitors	Refs.
Plasmepsins IX and X; PMIX, PMX (PF3D7_1430200, PF3D7_0808200)	Aspartic proteases that regulate the maturation of enzymes required to disrupt host cell membranes during parasite egress	WM382	77,201–203
Serine hydroxymethyltransferase; SHM (PF3D7_1456100)	Converts serine to glycine	-	204
Glycylpeptide *N*-tetradecanoyltransferase; NMT (PF3D7_1412800)	Transfers myristate to the N-terminal glycine of several protein molecules	IMP-1002	205,206
Lysyl tRNA synthetase; KRS (PF3D7_1350100)	ATP-dependent enzyme that catalyzes charging the tRNA synthetase with lysine; essential for protein biosynthesis	-	101
Acyl-CoA synthetases; ACS10/11 (PF3D7_0525100, PF3D7_1238800)	Coenzymes predicted to be involved in the synthesis of long-chain fatty acids	GSK701	92,207,208
Acetyl-CoA synthetase; AcAS (PF3D7_0627800)	Involved in the synthesis of acetyl-CoA	MMV019721, MMV084978	91
Cyclin-dependent-like kinase 3; CLK3 (PF3D7_1114700)	Kinase involved in mRNA splicing; inhibition results in lethal defects in mRNA processing	TCMDC-135051	114
cGMP-dependent protein kinase; PKG (PF3D7_1436600)	Involved in cyclic GMP signalling; an essential protein	MMV030084	94
Tyrosine tRNA synthetase; YRS (PF3D7_1117500)	ATP-dependent enzyme that catalyzes charging tRNA synthetase with tyrosine; essential for protein biosynthesis	ML901	105
Phenylalanine tRNA synthetase; FRS (PF3D7_0109800)	Catalyzes charging tRNA synthetase with phenylalanine	BRD5018	209
Threonine tRNA synthetase; TRS (PF3D7_1126000)	Catalyzes charging tRNA synthetase with threonine; essential for protein biosynthesis	Borrelidin	210
Histone deacetylase 1; HDAC1 (PF3D7_0925700)	Removes acetyl groups from histones	JF363, JX35	211–213
Proteasome (including PF3D7_1011400)	A multisubunit protein complex that drives protein degradation	Vinyl sulfones, epoxyketones, macrocyclic peptides, asparagine ethylenediamines	76,117,119,122,214,215

**Table 3 |:** Antimalarial targets with in vitro validation

Target (systematic name)	Mechanism of action	Noteworthy inhibitors	Refs.
Farnesyl pyrophosphate–geranylgeranyl diphosphate synthase; FPPS–GGPPS (PF3D7_1128400)	Dual-function enzyme involved in prenylation of C15 and C20 chains	MMV019313	216–221
Niemann–Pick type C1-related protein; NCR1 (PF3D7_0107500)	Transports lipophilic substrates and recycles sphingolipids; essential for maintaining the membrane lipid composition of blood-stage parasites	–	222
GCN5 (PF3D7_0823300)	Histone acetyltransferase	-	223
Dephospho-CoA kinase; DPCK (PF3D7_1443700)	Required for coenzyme A synthesis in the apicoplast	-	207,224,225
Adenylyl cyclase-β; ACβ (PF3D7_0802600)	Synthesizes the second messenger cAMP	-	226
Hexose transporter 1; HT1 (PF3D7_0204700)	Transports essential hexose across the parasite plasma membrane	-	227
cGMP-specific 3′,5′-cyclic phosphodiesterase-δ; PDE (PF3D7_1470500)	Expressed by mature gametocytes and regulates erythrocytic mechanical properties	Tadalafil	228
2-C-methyl-d-erythritol 4-phosphate cytidylyl; IspD (PF3D7_0106900)	Acts in the methylerythritol phosphate pathway, which synthesizes the essential isoprenoid precursors isopentenyl diphosphate and dimethylallyl diphosphate	MMV008138	229,230
Formate-nitrite transporter; FNT (PF3D7_0316600)	A high-capacity lactate-proton symporter	-	231–235
Hypoxanthine-guanine phosphoribosyltransferase; HGXPRT (PF3D7_1012400)	Essential enzyme in the purine salvage pathway	-	236
Purine nucleoside phosphorylase; PNP (PF3D7_0513300)	Essential enzyme in the purine salvage pathway	-	115
Malate:quinone oxidoreductase; MQO (PF3D7_0616800)	Essential enzyme in mitochondrial electron transport, the tricarboxylic acid cycle and the fumarate cycle	-	237
CPSF (PF3D7_0513200)	Regulates mRNA polyadenylation	AN3661	238
Monoacylglycerol lipase; MAGL (PF3D7_1038900)	Mediates hydrolysis of lipid esters, including a MAGL acylglycerol substrate	Salinopostin A	239
Leucine–tRNA ligase; LRS (PF3D7_0622800)	Essential enzyme that catalyzes charging tRNA synthetase with phenylalanine	Benzoxaboroles	104
Isoleucine–tRNA ligase; cIRS (PF3D7_1332900)	Essential enzyme in blood and liver stages	Thienopyrimidines	106

**Table 4 |:** Approaches to antimalarial target discovery

	No tool compound	Tool compound available			
Methods	Genomic and homology methods	In vitro evolution and whole-genome analysis	Proteomic methods	Overexpression or knockdown libraries	In silico methods
Advantages	Does not involve chemical tool compound; can give large numbers of candidate targets (but possible false positives)	Has led to dozens of targets in malaria; does not require many upfront biological tools	Has worked a few times for *Plasmodium;* does not require many upfront biological tools	Has worked well in other species	Lower cost
Disadvantages	Extensive downstream work required; high risk of failure	Can be time-consuming; can give resistance genes; might not work for all compounds	Might not work for all compounds; might give false positives; might miss important proteins	Requires development of biological tools; can give resistance genes; might give false positives	Limited accuracy; not good for compounds with novel mechanisms; difficult to test hypotheses; extensive downstream work required
Examples of targets	GCN5, GGPPS, DHODH	PI4K, KRS, YRS, FRS, PRS, CytB, EF2, ACS, PfATP4, GGPPS, YRS DHODH, DHFR-TS	PI4K, PKG	None to date, but good examples from other species	DHFR, DHODH, CytB

**Table 5 |:** Decision criteria for advancing a new chemical entity from an early lead to the preclinical stage

	Early lead	Late lead	Preclinical candidate
Potency/efficacy	EC_50_ (abs) <100 nM; SI >100×; profiled on liver stage, gametocytes and field isolates *(Plasmodium falciparum* and *Plasmodium vivax);* no cross-resistance with antimalarial drug or clinical candidate; MIR >10^6^; in vivo efficacy in NSG *P. falciparum* mouse model	EC_50_ (abs) <10 nM; SI >1,000×; profiled against *P. falciparum* SMFA; in vivo efficacy in NSG mouse model and data modelled (Medicines for Malaria Venture pharmacometrics) to estimate in vivo EC_50_; single oral dose prediction <500mg	Resistance risk defined; sequencing of in vivo recrudescent parasites
Pharmacokinetic properties	Solubility >100 μM; PK in mouse and rat: oral bioavailability >20%; in vitro metabolism (stability) predicting in vivo PK	Solubility >100 μM; PK in mouse, rat and dog: oral bioavailability >30%; human PK prediction; crystalline formulation used in PK studies	CYP450 inhibition and induction assays, CYP phenotyping, metabolite assays; full physical property and DMPK package; high dose PK in dog to inform for GLP safety studies
Safety	No overt toxicity in vivo and hERG >1 μM	Free hERG IC_20_/free C_max_ >30; negative in five-strain Ames assay; acceptable phototoxicity risk; acceptable off-target pharmacology profile (100 screens); measured in vitro reprotoxicology assays	No haemolysis in NSG mouse model engrafted with low active G6PD-deficient human blood in vivo; negative in micronucleus assay; acceptable margin in 7-day rat safety study; acceptable margin in in vivo early embryo development study

C_max_, highest concentration of a drug in the blood, cerebrospinal fluid or target organ after a dose is given; CYP450, cytochrome P450; DMPK, drug metabolism and pharmacokinetics; EC_50_ (abs), half-maximal effective concentration (asexual blood stages); G6DP, glucose-6-phosphate dehydrogenase; GLP, Good Laboratory Practices; hERG, human ether-a-go-go-related protein; IC_20_, concentration of a substance showing 20% inhibition relative to the maximum enzyme activity. MIR, minimum inoculum of resistance (minimum inoculum from which resistant parasites can be selected^64^); NSG, NOD–SCID–IL2Rγ^null^; PK, pharmacokinetics; SI, selectivity index; SMFA, standard membrane feeding assay.

## Box 1 PDPs in antimalarial drug discovery and development

Malaria mainly impacts populations in low-income and medium-income countries and therefore presents a low commercial incentive for pharmaceutical companies to obtain a return on their investment. Product development partnerships (PDPs) have been established to overcome this market impediment. The model is based on partnerships between public, private, academic and philanthropic sectors and is coordinated and operated by non-profit, virtual research and development organizations. The virtual aspect is based on the principle that much of the laboratory and clinical work is performed through partners, external research facilities and contractors, whereas the organization itself is responsible for the management, scientific overview and strategic oversight of the research programmes. The Medicines for Malaria Venture (MMV) is a PDP focused on discovery, development and delivery of new antimalarials. Other examples of successful PDPs are the Drugs for Neglected Diseases initiative, TB Alliance, Foundation for Innovative New Diagnostics (FIND), Innovative Vector Control Consortium (IVCC), European Vaccine Initiative (EVI) and International Vaccine Institute (IVI), with varying scopes of activities, including the development of drugs, vaccines, diagnostics and vector control solutions.

PDPs can also be more efficient than traditional pharmaceutical companies. The estimated investment necessary to bring a new drug to market is up to US$2.8 billion, based on data from 2009 to 2018 (ref. 241). By contrast, the Drugs for Neglected Diseases initiative (DNDi) reports an investment of $70–225 million to bring a new chemical entity to the market, and $7–38 million to improve existing drugs, thereby demonstrating an extremely cost-effective process compared with traditional pharmaceutical industry development. The funding for PDPs is provided upfront from philanthropic organizations, through in-kind donations from pharmaceutical companies and from public sources such as governments. This funding mechanism does not depend on borrowing capital; therefore, interest payments are avoided to reduce costs.

Another unique aspect of PDPs is that they allow industry ‘competitors’ to collaborate. MMV launched the Malaria Drug Development Catalyst, which provides a legal and scientific framework between industry partners to accelerate the development of next-generation antimalarial drug combinations. PDPs work simultaneously on multiple projects, following best practices on portfolio management from industry.

Overall, the success of PDPs relies on a combination of factors, including sustainable and robust funding mechanisms with a product instead of profit as the goal, lean operations, synergism instead of competition between partners, scientific and industry expertise, and efficient decision-making processes. To maximize the chances of success, PDPs also engage local communities, including researchers, care providers, policymakers, advocates and decision-makers, as stakeholders to guarantee ideal conditions.

MMV, with its partners, has delivered multiple products, including tafenoquine^242^ with GSK, which became the first new treatment in 60 years and the first single-dose treatment for *Plasmodium vivax* malaria targeting the liver stage parasites, including hypnozoites. More about PDPs can be read in the *Keeping the Promise* report^243^. MMV is also a member of the Malaria Drug Accelerator (MalDA) Consortium, a group of 18 laboratories, research organizations and companies that coordinates the discovery of early leads.

## Box 2 Alternative approaches to malaria therapy

### Monoclonal antibodies (mAbs)

Using mAbs to target pre-erythrocyte stages so that parasites are inhibited before they reach the liver is showing promise^244^. The ability of sporozoites to invade and infect hepatocytes relies on the circumsporozoite protein (CSP)^245^. Inducing an immune response against CSP is the mechanism of several advanced malaria vaccines^246^. Encouragingly, a modified CSP-specific mAb (CIS43LS) was tested in phase I^247^ and phase II^248^ clinical trials and showed efficacy of up to 88.2% over 6 months without evident safety concerns. L9LS, a next-generation antimalarial mAb, was approximately three times more potent than CIS43, the parent antibody of CIS43LS, in preclinical models^249^. A phase I clinical trial with L9LS demonstrated a half-life of almost 2 months and 88% protection after controlled human malaria infection, without evident safety concerns^135^. Other mAbs for malaria prophylaxis include MAM01 (ATRC-501; Gates Medical Research Institute) and TB-31F, which targets Pfs48/45, a gametocyte surface protein critical for parasite development and transmission^250^. Investing in the widespread use of mAbs is currently unaffordable for low-income and medium-income countries but could be cost-effective in some markets where health care and hospital stays are much more expensive, especially when no vaccines are available^251^.

### Long-acting injectables

The development of long-acting injectables requires considerations such as their safety profile, the *Plasmodium* target and degree of protection against resistance^252^, the potency as well as physical and pharmacokinetic properties of the drug, potential drug combinations and patient requirements. Moving to a once-per-season injectable regimen for prophylaxis would also require a new regulatory and clinical approach. Although there have been limited clinical studies, a target product profile for an injectable medicine providing long-term malaria protection has been proposed^253^. Furthermore, nanomaterials, including liposomes, polymeric nanoparticles or dendrimers, have been suggested as effective antimalarial drug delivery vehicles at optimized doses in injectable formulations^254^. Examples of potential long-acting injectables are ELQ-300 and ELQ-331 (ref. 166).

### Targeting mosquito vectors

Vector control is considered by the WHO to be a highly effective way to reduce malaria transmission. Long-lasting insecticidal nets have been applied for decades with both successes and challenges, including mosquito resistance^255^. Exposing mosquitoes to antimalarial drugs can be also effective. When female *Anopheles gambiae* take up low concentrations of atovaquone, parasite development is rapidly arrested in the insect midgut, preventing transmission^256^. Treating the mosquito with antimalarials is effective against artemisinin-resistant parasites and insecticide-resistant mosquitoes, representing a valuable approach towards malaria control and elimination^257^. There has also been significant progress in genetically modifying mosquitoes so that they are refractory to parasites^258^ or to reduce the mosquito population^259^. In addition, the use of endectocides (TCP-6) is a promising approach^260^ involving systemic treatment with long-lasting insecticides such as ivermectin, lotilaner or an isoxazoline^261,262^.

### Immunomodulators

The kinase inhibitor ruxolitinib is a modulator of the host interferon response and is being tested in combination with antimalarial therapy to modulate the long-term host immune response.

## Box 3 Relapsing forms of malaria

Two species of malaria parasite, *Plasmodium vivax* and *Plasmodium ovale*, form hypnozoites and can remain dormant for weeks to years in the liver cells of individuals with the infection. For reasons yet to be understood, the parasite reawakens and begins replicating again, eventually resulting in symptomatic blood-stage infection and further disease transmission.

Treating a population with a ‘radical cure’ drug is more difficult than it seems. Radical cure is defined by elimination of all parasites, including hypnozoites. There is only one compound class (8-aminoquinoline) effective against liver hypnozoites. This class includes primaquine and the recently approved primaquine analogue tafenoquine (Krintafel)^263,264^. Primaquine needs to be taken for at least a week, whereas tafenoquine has improved pharmacokinetic properties that allow single-dose administration^264^. The mechanism of action of the compounds is obscure, although they might trigger apoptosis of the infected liver cell^265^. Little is known about parasite resistance to 8-aminoquinolines. Some people are unable to metabolize primaquine and, because the active molecule is a metabolite^266^, the drug is not effective for these people^267^. The problems associated with 8-aminoquinolines have prompted extensive searches for replacements. Primaquine was discovered in the 1940s using an empirical approach: hundreds of *Rhesus* macaques were given *P. vivax* malaria and various antimalarial compounds and were then monitored for relapse. Although monkey-based screening assays are no longer practical and ethical, cellular phenotypic screening assays have been developed that could reveal compounds with potential radical cure activity. In these assays, compounds are added to liver cell cultures^268,269^ and then parasite types (replicating versus non-replicating) and numbers are counted using microscopy several days after sporozoite addition. These phenotypic screening methods are expensive, laborious and somewhat impractical. *P. vivax* blood-stage parasites cannot be maintained in blood-stage culture in the same way that *P. falciparum* can, necessitating the recruitment of infected human volunteers, who can be difficult to find near major research centres. For liver cell infection, a colony of mosquitoes is needed to produce sporozoites for the assay and there are logistical issues associated with rearing semi-domesticated species of mosquitoes in countries where they would thrive if released inadvertently.

Problems such as obtaining parasite material for phenotypic assays would dissipate if a target for a radical cure drug could be identified. Unfortunately, none are yet known and work to discover possible targets has lagged owing to the lack of material and because many of the known methods for target identification of phenotypic screening hits are unlikely to work. It is also possible that 8-aminoquinolines work via human targets in the infected liver cell. Work on rodent parasites shows that the parasite promotes massive host cell remodelling in the liver stage. Hopefully, as the biology of liver stages is further elucidated, including the importance of host pathways, a breakthrough discovery is on the horizon.
